# Medical and Philosophical News

**Published:** 1781-03

**Authors:** 


The life of the celebrated Linnæus, written by Dr. Poulteney, F. R. S. Phyſician at Blandford, is in the preſs, and will ſpeedily be publiſhed.

